# Supplementary material for: Immune DNA signature of T-cell infiltration in breast tumor exomes
Source: Sci Rep. 2016 Jul 25;6:30064. doi: 10.1038/srep30064 (PMC4958917; doi:10.1038/srep30064)
Supplement: Supplementary Information [file srep30064-s1.pdf]

Supplementary Information for Levy et al.

Immune DNA signature of T-cell infiltration in breast tumor exomes

Eric Levy, Rachel Marty ,Valentina Garate-Calderon, Brian Woo, Michelle Dow, Ricardo Armisen, Hannah Carter, Olivier Harismendy

Supplementary Tables S1-S5 are available online

Supplementary Figure S1-S3 are inserted below

a

Clone Frequency	Number of Clones			
	**OX4015F**	**OX1304F**	**OX1285F2**	**OX1285F1**
**High (>1%)**	4	1	3	3
**Medium (0.1%-1%)**	132	56	102	107
**Low (<0.1%)**	4,056	6,020	6,278	5,525
**Singleton (N=1 read)**	10,739	16,770	23,428	20,058
**Total**	14,931	22,847	29,811	25,693

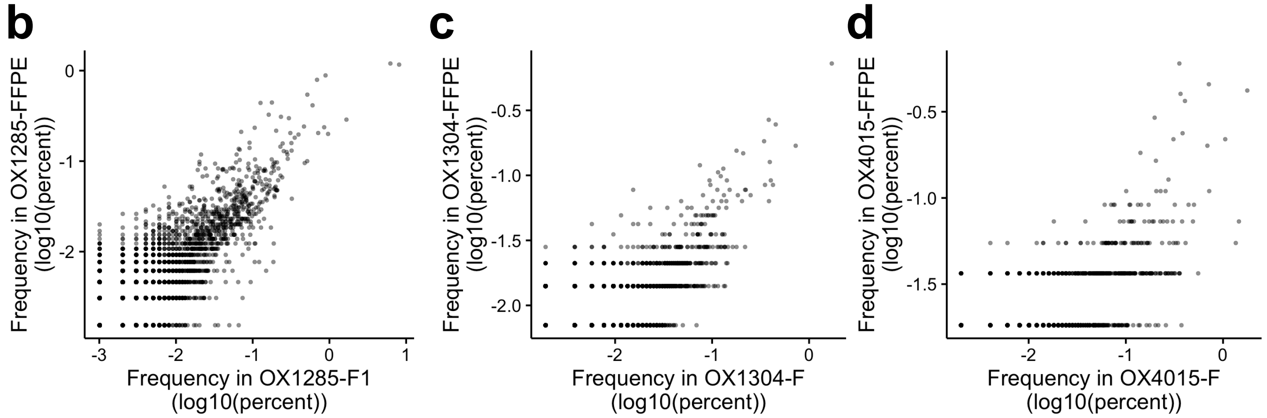

Supplementary Figure S1: Deep Repertoire Sequencing by Adaptive ImmunoSeq. (a) Distribution of clone frequency in the four specimen studied. **(b-d)** Scatter plot of clonotype abundance for mirrored frozen and FFPE tissue sections for samples OX1285 (b), OX1304 (c), OX4015 (d).

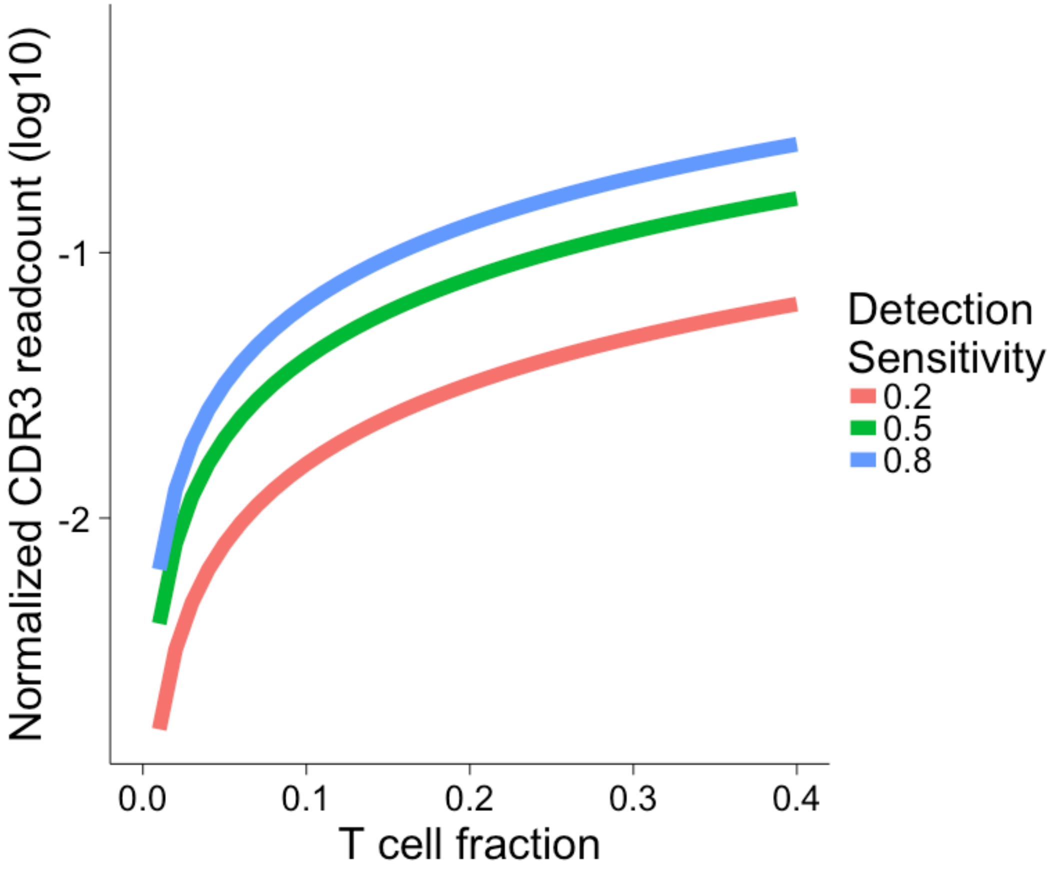

Supplementary Figure S2: Read detection simulation for CDR3 reads in whole-exome data. Simulated fraction of CDR3 reads (y-axis) expected from a whole-exome sequencing experiment, as a function of T-cell fraction (x-axis) and CDR3 detection efficiency. See methods for details.

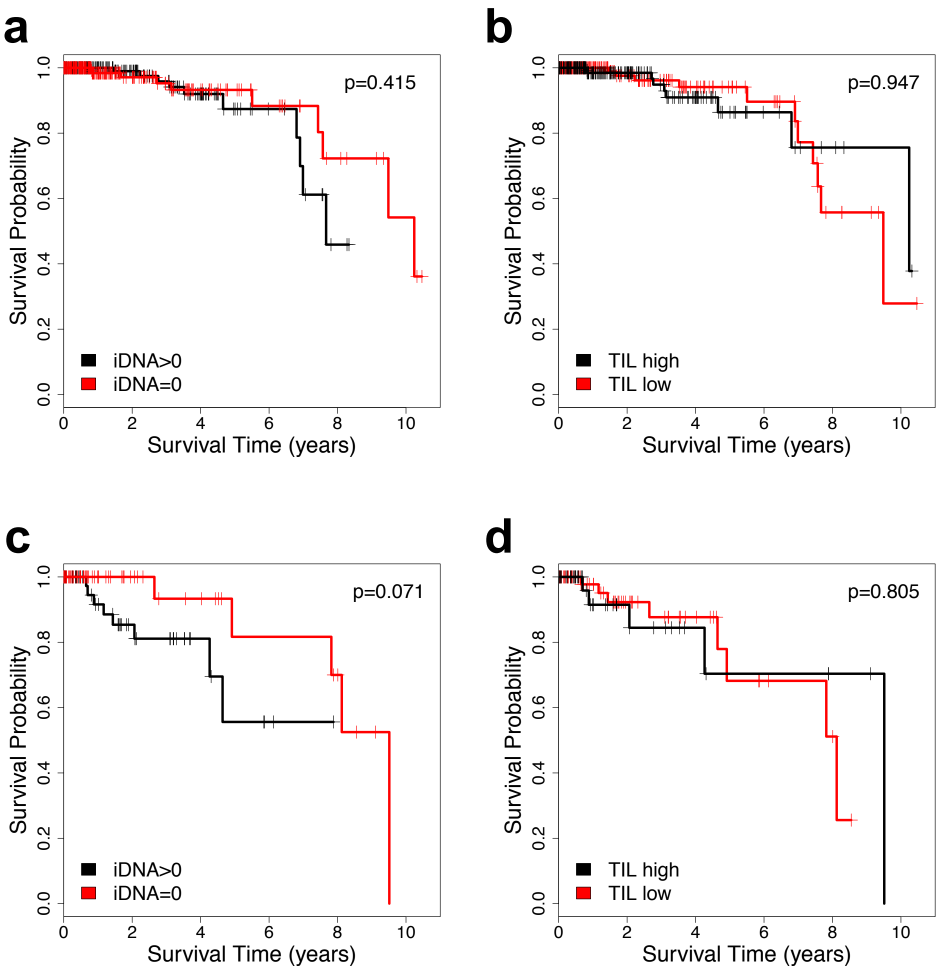

Supplementary Figure S3: BRCA subtype iDNA and survival analysis. Kaplan-Meier survival analysis with significance of the hazard ratio of (**a**) HR+ patients as a function of iDNA score. Hazard-ratio is 0.676 [0.264-1.73]. **(b)** HR + patients as a function of TILs. Hazard-ratio is 1.03 [0.420-2.53]. **(c)** TNBC patients as a function of iDNA score. Hazard-ratio is 0.277 [0.069-1.12]. **(d)** TNBC patients as a function of TILs. Hazard-ratio is 0.859 [0.256-2.88].
